# A study of the distribution of phylogenetically conserved blocks within clusters of mammalian homeobox genes

**DOI:** 10.1590/S1415-47572009000300034

**Published:** 2009-09-01

**Authors:** Nikola Stojanovic

**Affiliations:** Department of Computer Science and Engineering, University of Texas at Arlington, Arlington, TexasUSA

**Keywords:** genomics, phylogenetic conservation, multiple sequence alignments, heaviest segments, Hox

## Abstract

Genome sequencing efforts of the last decade have produced a large amount of data, which has enabled whole-genome comparative analyses in order to locate potentially functional elements and study the overall patterns of phylogenetic conservation. In this paper we present a statistically based method for the characterization of these patterns in mammalian DNA sequences. We have applied this approach to the study of exceptionally well conserved homeobox gene clusters (*Hox*), based on an alignment of six species, and we have constructed a map of *Hox* cataloguing the conserved fragments, along with their locations in relation to the genes and other landmarks, sometimes showing unexpected layouts.

## Introduction

The power of comparative analysis of genomic sequence data has been recognized for many years. The conservation patterns among related species reveal the homology between genomic segments, as well as the effects of functional constraints on mutations (Hardison, 2000; Zody, 2007). It is generally understood that conserved regions did not succumb to the evolutionary drift due to the effect of deleterious mutations, and consequently sequence alignments became important for locating functional loci in DNA (Miller *et al.*, 2004). For larger regions, such as gene exons, even pairwise alignments are already sufficient if the target species are chosen well, but for the detection of subtler signals one needs multiple sequences. Until several years ago large scale studies of multiple alignments were not feasible in eukaryotes, mammals in particular, but with the advancement of sequencing projects this situation has dramatically changed. In particular, projects directed towards targeted sequencing of genomic regions for the purpose of the analysis of conservation (Thomas *et al.*, 2003; The ENCODE Project Consortium, 2004; Margulies *et al.*, 2005) have already produced substantial results (The ENCODE Project Consortium, 2007; King *et al.*, 2007).

Whereas the early attempts to identify functional DNA elements based on phylogenetic conservation were largely heuristic, there were continuous efforts to statistically characterize them with respect to the background (Li and Miller, 2002; Ganley and Kobayashi, 2008). Local background may amount to more than just a reference framework, as it has been known for more than a decade now that regions such as *Hox* gene clusters may be protected from evolutionary drift by some yet unknown mechanism (Duret and Bucher, 1997). Thus, if a region of good conservation appears unlikely in its environment that could be a strong indication that it is important. A region of a more ordinary composition can still be functional, but it does not stand out clearly enough to suggest function based solely on the conservation, so other methods may be needed (for instance, a combination of positional information with lookups in the databases of known motifs, such as TRANSFAC (Wingender, 2008) or Jaspar (Bryne *et al.*, 2008)). However, before addressing this issue one needs to characterize what “local” means. Concerning the rate of change between homologous sequences it should clearly be an area in which this rate does not vary much, overall.

The approach described in this paper is based on this local environment concept. We have applied it to the analysis of clusters of mammalian homeobox genes, also known as *Hox*. We have chosen them because they have been extensively studied, so the locations of genes and many other elements are well known, and also because of their good overall phylogenetic conservation which involves features other than simple sequence motifs, including the stability of intergenic spacing in orthologous, but not paralogous, clusters and an apparent resiliency to rearrangements and the insertion of transposable elements (International Human Genome Sequencing Consortium, 2001). There are four *Hox* clusters in mammals: *HoxA* on human chromosome 7, *HoxB* on chromosome 17, *HoxC* on chromosome 12, and *HoxD* on chromosome 2. They span about 100-200 kb each, and contain a total of 39 genes in human, divided in 13 groups of paralogs (labeled 1 through 13). *Hox* genes are ordered in the same way in each cluster, although not every cluster contains the full set of 13 genes. The function of the paralogs is only partially redundant, as the loss of one generally cannot be completely compensated by the others (Horan *et al.*, 1995; Duboule, 2000; Lynch *et al.*, 2006).

All *Hox* genes code for transcription factors which regulate the formation of the anterior-posterior axis of an animal during early embryonic development, acting on a large number of downstream genes (Foronda *et al.*, 2008, Li-Kroeger *et al.*, 2008). Since this axis is common throughout the evolution, *Hox* clusters are well conserved, often over regions much longer than expected under a simple model of coding sequence and transcriptional regulation. These genes are also triggered in a strict succession which corresponds to their spatial arrangement (Cobb and Duboule, 2004), so their order, and not just their sequence, appear to be under constraint. This was instrumental for our purpose, because we could build reliable multiple alignments, and also expect that the nature and positions of functional elements could not have varied much. However, the main goal of our work was the characterization of the overall phylogenetic sequence conservation in *Hox* clusters, rather than a search for individual functional elements. The latter task can be better achieved by projects such as ENCODE, where *HoxA* was one of its target regions during its pilot phase (The ENCODE Project Consortium, 2004). Many other groups are currently looking at *Hox* clusters, too, since developmental regulation is a subject of intensive research.

## Materials and Methods

As the first step in our analysis, we have constructed long alignments of all four *Hox* clusters and their surrounding regions (of about 500 kb each, measured in human sequence) from six mammals representing three distinct groups: two primates (human and baboon), two ungulates (cow and pig) and two rodents (mouse and rat), using Multi-LAGAN software (Brudno *et al.*, 2003). Since this alignment has been initially built for the targeted study of other genomic features, in an unrelated work by our collaborators, it included only 27 out of 39 *Hox* genes in its high-quality section, which has nevertheless provided us with long contiguous regions of about 90 kb of *HoxA* cluster, 150 kb of *HoxB* cluster, 160 kb of *HoxC* cluster and about 80 kb of *HoxD* cluster. These sequences totaled to about half megabase of *Hox*, comprising about half of the total area of the clusters and two thirds of the genes, so we considered these data sufficient for our study.

We then fragmented the alignments into large blocks where the conservation rate could have been considered constant. This was done iteratively, expanding small seed blocks until the application of the Central Limit Theorem indicated that the neighboring ones were unlikely to draw from the same distribution, at 0.99 or higher significance. The seed length has been set to 50, as we wanted it as short as possible, and below this minimal sample size the application of the CLT may have been unreliable. As a practical rule in statistics, it is a generally accepted that Central Limit Theorem should be used for sample sizes of at least 50, and preferably 100 or more. Below this count one can still use Student-*t* distribution with appropriate number of degrees of freedom, however special considerations concerning the underlying random variable must be observed.

The columns of the alignment were scored using our implementation of the weighted parsimony algorithm (Sankoff and Cedergren, 1983). However, we have tried to avoid introducing an arbitrary bias (since the relative evolution rates of the species we have considered are still largely unknown) and thus applied the uniform mutation costs, *i.e.* the unweighted scoring scheme, except for favoring transitions over transversions when there was a choice. For example, it has been well established that rodent evolution rate is faster than that of primates (Mouse Genome Sequencing Consortium, 2002), but even the relative positions of rodent and ungulate branches on the evolutionary tree, with respect to primates, are still somewhat controversial (since all three groups are at about equal distance of 80-100 Myr). In this study we have applied a model under which rodents are closer to primates, as there appears to be accumulating evidence in support of this hypothesis. Insertions and deletions, reflected as gaps in the alignment, were treated as any other substitutions, even if a chain of gaps likely corresponds to a single evolutionary event. This way the entire alignment could have been represented as an array of scores *s*_*i*_, divided into blocks of the initial seed length, and subject to further refinement. In each iterative step we have calculated the means μ and sample variances σ^2^ of the neighboring regions, then used the μ of the larger sample as the true mean, and the smaller sample for the calculation of the confidence interval. These steps were repeated until there was no change in the total number of blocks. Once it has been determined that neighboring blocks were unlikely to feature the same conservation rate, further refinement was performed in order to establish the most likely boundary, by adjusting it until it optimally distributed the columns closer to one of the two means.

It is intuitive that large blocks of relatively constant conservation should correspond to genome loci featuring the same mutation rates. This can be due to different concentration of long and short functional DNA elements, inducing varying degrees of constraint, or due to some other mechanism protecting specific domains. The latter, as a hypothesis, have been circulated among scientists for quite some time, however by now no such mechanism has been identified (except for the general repair capabilities of DNA, which are not position-specific). Once such blocks have been determined, we proceeded to identify the outliers. The expectation was that these outliers would roughly correspond to gene exons, as they would be the only known elements which would warrant relatively long blocks of consistent good correspondence.

After establishing the background conservation rate, it was possible to further isolate shorter regions significant within their own environment. Since the lower values for individual alignment column scores *s*_*i*_ obtained through the application of parsimony indicated better conservation, we modified them by subtracting *s*_*i*_ from the average local background divergence. That assigned the highest score to the most conserved columns, and only these scoring better than the local mean remained positive. However, we have now used an infinite negative score for gap-containing columns – while some significant areas might have been lost because of this strategy, it also protected us from dealing with blocks in which, for instance, all but one sequence featured a gap (in addition, a gap in any short region would likely preclude its function, and, furthermore, such gaps may even indicate just the lack of data, rather than a genuine evolutionary event). We have used the modified scores in order to isolate full runs of columns, also known as heaviest segments, by applying an algorithm (Stojanovic, 2009) we have adapted from Bentley (1986). Our implementation is technically different, but it produces the same effect as the algorithm of Ruzzo and Tompa (1999). We define the full runs as the maximal intervals cumulatively scoring higher than any of their subintervals.

Our algorithm locates all full runs in O(*n*) time, where *n* is the size of the score array, *i.e.* the number of columns in the alignment. In order to avoid the clutter and observe the trends, as opposed to individual sites, we have somewhat arbitrarily limited the minimal length of a considered full run to 25 columns. While this size may miss quite a few isolated transcriptional regulatory elements, it was appropriate for our goals, and for an environment conserved as well as *Hox* - other genomic regions may require a much smaller lower bound, although setting up an optimal one is more art than science.

We then calculated the mean and the variance for each of the located regions, and used these values for further comparisons. However, the located regions may still not be statistically significant in their own surroundings, so they needed to be examined in a more stringent way. Because they were generally short (up to a few dozen bases), we have used the Student-*t* test. Due to the decrease in variance when the average is taken over longer intervals (and the increase in the number of degrees of freedom), longer ones may be more likely to pass the significance threshold, and in a purely random setting they would be also less likely to stand out. This corresponds well with their presumed biological meaning, however the quality of the background conservation introduces a bias in the interpretation, relating to the well-known dilemma of whether a perfectly conserved block should be discarded simply because everything around it is well preserved. Blocks with a significant mean (*i.e.* these within outlying backgrounds, where everything which even slightly stands above the average is highly significant, in reality, if not by likelihood) should thus be considered by that measure only, while the statistical significance test should be applied to these discovered in poorer background conservation areas.

We have assumed that if the regions of constant conservation rates do not capture the exons of *Hox* genes throughout, at least their fragments should be recognized as long significant blocks. Shorter intervals may indicate possible transcription factor binding sites and other functional elements, but their actual prediction would require further work. The fact that a region is distinguished from its environment still need not imply that it has a function, or at least an obvious function, as recent experiments have demonstrated in a rather dramatic way (Ahituv *et al.*, 2007). We have thus limited our study to the annotation of the sequence sites according to how unusual they were, leaving the actual determination of their functionality to expert estimates, computational (using further evidence), and in the laboratory.

## Results

As soon as significant amounts of mammalian genomic sequences became available, including *Hox*, researchers started looking at large-scale synteny and other comparative features. This has led to the first, relatively informal, observations that the overall conservation patterns in alignments of some genomic segments, ours as well as these of other investigators, in *Hox* (Sabarinadh *et al.*, 2004) and elsewhere in mammalian genomes (Rijnkels *et al.*, 2003), did not appear to correlate well with the expectations. If an alignment is biologically correct (and a mathematical optimum under a good scoring scheme would presumably come close to that), one would expect that gene exons would stand out more-or-less clearly, while the regulatory sequences would be dotted with clusters of conserved transcription factor binding motifs. Because of the inter-species genetic variations and the lack of DNA sequence specificity of most regulatory proteins this rarely happens in reality, but a reasonably close alignment layout is intuitive (Stojanovic *et al.*, 1999).

Discarding the opening and closing gaps in incomplete sequences, for this analysis we have selected only these parts of our alignments which exhibited reasonable sequence and layout quality throughout. In *HoxA* that was from the second exon of *HoxA11* gene through about 11 kb 3' to *HoxA1* gene, including the 3', but not the 5' end of the cluster. In *HoxB* it was from about 7 kb 3' to *HoxB13* to about 13 kb 3' to *HoxB4*, thus missing several genes at both 5' and 3' end of the cluster. Due to the deactivation of three genes between *HoxB13* and *HoxB9* this left us with a large intergenic region at the opening end, which was beneficial for our study (Figure 2). In *HoxC* we have selected the area between about 48 kb 5' to *HoxC13* to about 15 kb 3' of *HoxC5*. This included the 5' end of the *HoxC* cluster, with a significant starting intergenic area, but excluded its 3' end, missing the *HoxC4* gene. In *HoxD* we had the least sequence to work with – our fragment included the last 264 bases of the intron of *HoxD4* (thus missing six *Hox* genes, plus one exon) until about 46 kb 3' of *HoxD1*. At the 3' end of the *HoxD* cluster we thus had a large segment of intergenic sequence, however there has been an Ensembl (Flicek *et al.*, 2008) prediction of another gene (XP_496612.1) in that area (a record which has been removed on re-annotation, despite the mRNA and EST evidence). Overall, this gave us a good blend of *Hox* environments in which any patterns should be clearly visible.

We were somewhat surprised by the outcome of the initial breakup of the alignments into areas of constant conservation rate, as they became divided into a large number of blocks of 250 bp on average, which could not have been merged further. This was primarily due to very low sample variances, and that confirmed the known fact that genomic sequences are far from random, even outside genes. However, since intervals of this size can capture exons, we were content with this kind of division, especially as it was stable, *i.e.* it did not substantially change with large increases in the significance threshold. Using this division we have also located shorter full runs standing out in these environments.

**Figure 1:**
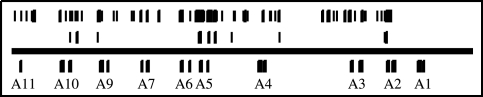
Sequence conservation in *HoxA* cluster. Thick horizontal line indicates the range which has been analyzed, with position of *HoxA* genes indicated below it (all *Hox* genes have two exons). The bars right above the line indicate the positions of larger (50-400 bp) environments conserved at, or below, 0.1 average parsimony level, and the bars above them indicate the positions of shorter (25-100 bp) regions at the same level of conservation.

First we considered all segments, either large constant-rate environments or shorter regions of minimal length 25 bp, featuring a parsimony score of at most 0.1 substitutions per site. Minimal length was set at 25 because it was unlikely that an individual element would be this long (with transcription factor binding sites of 5-25 bp, and miRNAs of about 22 bp), and we intended to analyze the trends only. The distribution of these areas in all four *Hox* clusters is shown in Figures 1 through 4. As it can be seen from these figures, the layout of these regions was slightly indicative of the concentration at the anterior end of the clusters, and some studies have indeed concluded that the mechanisms of regulation may be considerably different between groups of *Hox* genes, and that *cis*-acting elements were more likely to be found in the close proximity of the anterior genes, with posterior ones being regulated in increasingly complex and spatially distant ways (Sharpe *et al.*, 1998).

Because it is difficult to see from the figures where these regions were exactly located, we have tabulated their distribution over several distinct genomic domains, including 5' regulatory regions, exons, introns, 3' sequences and intergenic sequences, in Table 1 (counting the long regions) and Table 2 (short outlier regions). As we have mapped the *Hox* genes by the beginnings of their coding sequences, and in *Hox* they are always located in the first exon, the immediate 5' area contained the untranslated regions, with the promoter and the associated elements being more distant. Because of the varying sizes of the considered domains (for instance, the total length of all exons was much shorter than the intergenic areas), the absolute high conservation region counts were not very informative, so we have also measured the percentage of the columns contained in the regions with the mean less than 0.1, and shown the results in Table 3. Alternatively, we could count just the percentage of individual high scoring columns – this approach would likely yield similar results, but it could be swayed by the noise created by isolated instances.

**Figure 2:**
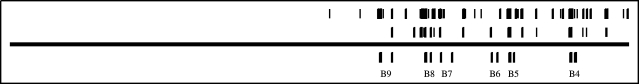
Sequence conservation in *HoxB* cluster. Thick horizontal line indicates the range which has been analyzed, with position of *HoxB* genes indicated below it (all *Hox* genes have two exons). The bars right above the line indicate the positions of larger (50-400 bp) environments conserved at, or below, 0.1 average parsimony level, and the bars above them indicate the positions of shorter (25-100 bp) regions at the same level of conservation.

**Figure 3:**
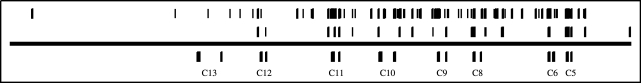
Sequence conservation in *HoxC* cluster. Thick horizontal line indicates the range which has been analyzed, with position of *HoxC* genes indicated below it (all *Hox* genes have two exons). The bars right above the line indicate the positions of larger (50-400 bp) environments conserved at, or below, 0.1 average parsimony level, and the bars above them indicate the positions of shorter (25-100 bp) regions at the same level of conservation.

The layout of these regions was somewhat surprising. It showed the highest density not in gene exons, as anticipated, but at their immediate 5' loci, normally containing the UTRs. This phenomenon has occasionally been noted by other studies, too, such as in *casein* gene clusters (Rijnkels *et al.*, 2003). The conservation density drops as one moves away from the genes, however the fact that the density has been measured over regions of minimal length 25 makes this observation somewhat puzzling. One can argue that some of these regions actually represent clusters of regulatory elements, since not every included column is required to maintain the same high conservation rate, but the overall conservation may still be too good for such scenario. It may also be that some are important for post-transcriptional regulation, and targets for micro-RNAs, known to be involved in directing *Hox* expression (Cobb and Duboule, 2004). However, from the substantial block conservation of the 5' UTRs and promoter regions one is indeed tempted to hypothesize that some yet unknown mechanism protects these entire areas from mutations, imposing a much wider constraint on the sequence than just on the functional elements.

Unexpectedly, no regions of high overall conservation have been found in the small part of the *HoxD* cluster we have analyzed. Further inspection has shown that both the exons of these genes and the corresponding 5' sequences were indeed conserved, although not at the stringent 0.1 substitution average level. More puzzling was the concentration of high conservation sites in what we initially considered an intergenic region, but they almost all lie at or around the site of the Ensembl gene prediction, providing additional evidence of an unusual situation at that locus. However, many highly conserved regions have been found in the intergenic regions of other *Hox* clusters, too. Some of them contain functional elements, although it is an open question why they were so long. A part of the explanation may be in the studies which have found miRNA genes within *Hox* clusters (Yekta *et al.*, 2004), and in that distal enhancer modules are common in the genome.

**Figure 4:**
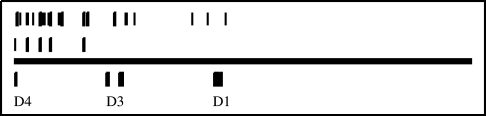
Sequence conservation in *HoxD* cluster. Thick horizontal line indicates the range which has been analyzed, with position of *HoxD* genes indicated below it (all *Hox* genes have two exons). The bars right above the line indicate the positions of larger (50-400 bp) environments conserved at, or below, 0.1 average parsimony level, and the bars above them indicate the positions of shorter (25-100 bp) regions at the same level of conservation.

## Discussion

In this paper we have presented findings which appear to further support an argument that the functional constraints on DNA sequences may be enforced by a mechanism broader than a simple prohibition of mutations within functional elements, even as no such mechanism has been discovered to date. The overall conservation patterns, both in the background and in the contiguous areas scoring better than the background indicate consistent good conservation in sequences upstream of the translation start sites, and often better than within the coding sequences themselves. In addition, a large number, if not a majority, of both long and short intervals which score better than their local environment do so with high significance.

**Figure 5:**
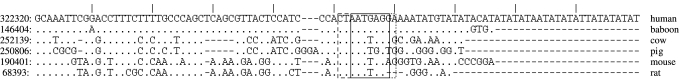
A region from *HoxA* cluster standing in a stark contrast with its environment. Solid line box encloses the area of perfect conservation in all species. Dashed and dotted lines show the areas of rodent and primate differential conservation, respectively. Dots indicate the same letter as in row 1 (human).

Although the goal of this work was to identify general trends only, rather than attempt to isolate short blocks and motifs with putative functional roles, our software was also capable of finding small isolated regions when they stood in a contrast with their environment (under a different parametrization scheme, though, permitting for much shorter outlier regions than considered in the rest of this study), as depicted in Figure 5. Here we have looked at the regions of minimal length five whose cumulative score was exceeding the mean of their environment. As expected, many such regions have been located, and we have again used the Student-*t* test to estimate their significance. Roughly half were significant above the 0.99 level, again confirming the fundamental non-random nature of genomic sequences. Some of these loci were clustering, but there was only an occasional match with experimentally confirmed functional sites (dataset compiled from the literature by Laura Elnitski, unpublished). As these regions were more likely to stand out only in the areas of poor general conservation, we have not attempted to plot them on a chart similar to these illustrating more global features of *Hox* (in Figures 1 through 4).

**Figure 6:**
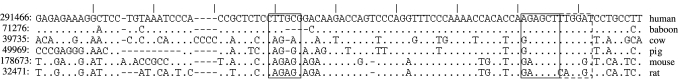
A region from *HoxC* cluster exhibiting several blocks of good differential conservation, otherwise not obvious within the environment. Solid and dashed lines indicate different kinds of differential conservation. Dots indicate the same letter as in row 1 (human).

We have also adapted our approach to look for otherwise difficult-to-spot areas where the compared species appear to exhibit different patterns of conservation (differential phylogenetic footprints (Gumucio *et al.*, 1994, Stojanovic, 2004), as illustrated in Figure 6. In this context, we have looked at short areas where sequences within a particular group agreed, but did not feature significant overall conservation between the groups. However, this required more elaborate modifications to the method reported in this manuscript, and will be a subject of another report.

Many other aspects of our methods are still a work in progress. We need to refine our ways of integrating data from different background conservation levels and long and short outlier regions, along with their significance, eliminating as many of the heuristics as it is possible in a genomic context. There are existing programs, such as Multi-PipMaker (Schwartz *et al.*, 2000), or more recent comparative genomics tools developed by the University of California at Santa Cruz Genome Browser team (Karolchik *et al.* 2008; Rosenbloom *et al.* 2008) and the efforts of The ENCODE Project Consortium (2007) which perform similar tasks, and usually provide an intuitive graphical representation, too. However, our approach is original, and it can be used in a complementary way with other software. Another possible extension of this work may involve the replacement of our custom-built alignments with large pre-made assemblies which are being increasingly made publicly available (Miller *et al.*, 2007). However, while nobody disputes the utility of multiple alignments involving sequences from dozens of related species, much smaller ones, such as our six-row construct, can serve well for the identification of overall conservational patterns. Using a very large number of species for this purpose may be an overkill, even if reliable deep pre-made alignments would be available for every genomic region under study.

It may be of interest to further divide the areas in which overall patterns of conservation are recorded to at least separate the exons of the considered genes (first exon as opposed to the rest), and then subdivide the first exon, when feasible, into the UTR and the transcribed part, and even at a finer granularity. The reason for that is in that the ENCODE consortium has reported (The ENCODE Project Consortium. 2007) that sites important for the initiation of transcription appear to be symmetrically distributed around the transcription start site, contrary to what has been previously thought. This may explain some of the exceptionally high conservation we have observed in the 5' UTR regions, although probably not all of it. However, one always has to keep in mind that these loci are important for many aspects of gene expression other than just the initiation of transcription, such as elongation and post-transcriptional regulation.

We also plan to perform further systematic analysis by varying thresholds for the mean score of the conservation, and analyze the trends. In addition, our treatment of gaps in sequences, while practical, is not satisfactory. The uncritical inclusion of gaps often leads to artifacts, but their exclusion creates problems, too. No matter how uncomfortable they are to work with, gaps in alignments are presumed to reflect the natural process of nucleotide insertion and deletion, and as such they should be fully considered in the analysis.

## Figures and Tables

**Table 1:** Number of long regions of average conservation of 0.1 substitution per site, or better, falling into each distinct genomic domain. The intergenic domain number for *HoxD* has been parenthesized because of the earlier Ensembl gene prediction at the location where many of the conserved regions have been found.

	500-1000 5'	200-500 5'	1-200 5'	Exons	Introns	1-1000 3'	Intergenic
*HoxA*	2	1	1	2	0	1	4
*HoxB*	1	2	5	9	0	1	10
*HoxC*	1	2	4	7	4	2	8
*HoxD*	0	0	0	1	0	0	(4)

**Table 2:** Number of short outlier regions of average conservation of 0.1 substitution per site, or better, falling into each distinct genomic domain. The intergenic domain number for *HoxD* has been parenthesized because of the earlier Ensembl gene prediction at the location where many of the conserved regions have been found.

	500-1000 5'	200-500 5'	1-200 5'	Exons	Introns	1-1000 3'	Intergenic
*HoxA*	5	10	14	28	10	15	54
*HoxB*	4	7	7	3	8	14	29
*HoxC*	3	4	9	8	13	15	51
*HoxD*	0	0	0	0	2	4	(22)

**Table 3:** Fractions of the total number of alignment columns in each distinct genomic domain contained in the regions of minimal length 25 bp, with average conservation rate of 0.1 or better. The intergenic data for *HoxD* have been parenthesized because of the Ensembl gene prediction at the location where many of these regions were found.

	500-1000 5'	200-500 5'	1-200 5'	Exons	Introns	1-1000 3'	Intergenic
*HoxA*	0.067	0.315	0.616	0.223	0.066	0.077	0.057
*HoxB*	0.115	0.342	0.788	0.639	0.071	0.145	0.024
*HoxC*	0.104	0.202	0.609	0.521	0.089	0.105	0.035
*HoxD*	0	0	0	0.061	0.026	0.066	(0.027)

## References

[Ahituvetal2007] Ahituv N., Zhu Y., Visel A., Holt A., Afzal V., Pennacchio L.A., Rubin E.M. (2007). Deletion of ultraconserved elements yields viable mice. PLoS Biol.

[Bentley1986] Bentley J.L. (1986). Programming Pearls.

[Brudnoetal2003] Brudno M., Do C.B., Cooper G.M., Kim M.F., Davydov E., Green E.D., Sidow A., Batzoglou S. (2003). Lagan and Multi-LAGAN: Effcient tools for large-scale multiple alignment of genomic DNA. Genome Res.

[Bryneetal2008] Bryne J.C., Valen E., Tang M.H., Marstrand T., Winther O., da Piedade I., Krogh A., Lenhard B., Sandelin A. (2008). JASPAR, the open access database of transcription factor-binding profiles: New content and tools in the 2008 update. Nucleic Acids Res.

[CobbandDuboule2004] Cobb J., Duboule D. (2004). Tracing microRNA patterns in mice. Nat Genet.

[Duboule2000] Duboule D. (2000). A hox by any other name. Nature.

[DuretandBucher1997] Duret L., Bucher P. (1997). Searching for regulatory elements in human noncoding sequences. Curr Opin Struct Biol.

[Fliceketal2008] Flicek P., Aken B., Beal K., Ballester B., Caccamo M., Chen Y., Clarke L., Coates G., Cunningham F., Cutts T. (2008). Ensembl 2008. Nucleic Acids Res.

[Forondaetal2008] Foronda D., de Navas L.F., Garaulet D.L., Sanchez-Herrero E. (2008). Function and specificity of genes. Int J Dev Biol.

[GanleyandKobayashi2007] Ganley A.R., Kobayashi T. (2007). Phylogenetic footprinting to find functional DNA elements. Methods Mol Biol.

[Gumucioetal1994] Gumucio D., Shelton D., Blanchard-McQuate K., Gray T., Tarle S., Heilstedt-Williamson H., Slightom J., Collins F., Goodman M. (1994). Differential phylogenetic footprinting as a means to identify base changes responsible for recruitment of the anthropoid γ gene to a fetal expression pattern. J Biol Chem.

[Hardison2000] Hardison R. (2000). Conserved noncoding sequences are reliable guides to regulatory elements. Trends Genet.

[Horanetal1995] Horan G., Kovacs E., Behringer R., Featherstone M. (1995). Mutations in paralogous *Hox* genes result in overlapping homeotic transformations of the axial skeleton: Evidence for unique and redundant function. Dev Biol.

[InternationalHumanGenomeSequencingConsortium2001] International Human Genome Sequencing Consortium (2001). Initial sequencing and analysis of the human genome. Nature.

[Karolchiketal2008] Karolchik D., Kuhn R., Baertsch R., Barber G., Clawson H., Diekhans M., Giardine B., Harte R., Hinrichs A., Hsu F. (2008). The UCSC genome browser database: 2008 update. Nucleic Acids Res.

[Kingetal2007] King D.C., Taylor J., Zhang Y., Cheng Y., Lawson H.A., Martin J., Chiaromonte F., Miller W., Hardison R.C., ENCODE groups for Transcriptional Regulation Multispecies Sequence Analysis (2007). Finding *cis*-regulatory elements using comparative genomics: Some lessons from ENCODE data. Genome Res.

[LiandMiller2002] Li J., Miller W. (2002). Significance of inter-species matches when evolutionary rate varies. Proceeding of the Sixth Annual International Conference on Computational Biology.

[Li-Kroegeretal2008] Li-Kroeger D., Lorraine Witt L.M., Grimes H.L., Cook T.A., Gebelein B. (2008). *Hox* and *Senseless* antagonism functions as a molecular switch to regulate EGF secretion in the *Drosophila* PNS. Dev Cell.

[Lynchetal2006] Lynch V.J., Roth J.J., Wagner G.P. (2006). Adaptive evolution of hox-gene homeodomains after cluster duplications. BMC Evol Biol.

[Marguliesetal2005] Margulies E., Vinson J., NISC Comparative Sequencing Program Miller W., Jaffe D., Lindblad-Toh K., Chang J., Green E., Lander E., Mullikin J. (2005). An initial strategy for the systematic identification of functional elements in the human genome by low-redundancy comparative sequencing. Proc Natl Acad Sci USA.

[Milleretal2004] Miller W., Makova K., Nekrutenko A., Hardison R.C. (2004). Comparative genomics. Annu Rev Genomics Hum Genet.

[Milleretal2007] Miller W., Rosenbloom K., Hardison R.C., Hou M., Taylor J., Raney B., Burhans R., King D.C., Baertsch R., Blankenberg D. (2007). 28-Way vertebrate alignment and conservation track in the UCSC Genome Browser. Genome Res.

[MouseGenomeSequencingConsortium2002] Mouse Genome Sequencing Consortium (2002). Initial sequencing and comparative analysis of the mouse genome. Nature.

[Rijnkelsetal2003] Rijnkels M., Elnitski L., Miller W., Rosen J.M. (2003). Multispecies comparative analysis of a mammalian-specific genomic domain encoding secretory proteins. Genomics.

[Rosenbloometal2008] Rosenbloom K., Taylor J., Schaeffer S., Kent J., Haussler D., Miller W. (2008). Phylogenomic resources at the UCSC Genome Browser. Methods Mol Biol.

[RuzzoandTompa1999] Ruzzo W., Tompa M. (1999). A linear time algorithm for finding all maximal scoring subsequences. Proceedings of the Seventh International Conference on Intelligent Systems for Molecular Biology.

[Sabarinadhetal2004] Sabarinadh C., Subramanian S., Tripathi A., Mishra R.K. (2004). Extreme conservation of noncoding DNA near hoxd complex of vertebrates. BMC Genomics.

[SankoffandCedergren1983] Sankoff D., Cedergren R., Sankoff D., Kruskal J. (1983). Simultaneous comparison of three or more sequences related by a tree. Time Warps, String Edits, and Macromolecules: The Theory and Practice of Sequence Comparison.

[Schwartzetal2000] Schwartz S., Zhang Z., Frazer K., Smith A., Riemer C., Bouck J., Gibbs R., Hardison R., Miller W. (2000). PipMaker - A web server for aligning two genomic DNA sequences. Genome Res.

[Sharpeetal1998] Sharpe J., Nonchev S., Gould A., Whiting J., Krumlauf R. (1998). Selectivity, sharing and competitive interactions in the regulation of Hoxb genes. EMBO J.

[Stojanovicetal1999] Stojanovic N., Florea L., Riemer C., Gumucio D., Slightom J., Goodman M., Miller W., Hardison R. (1999). Comparison of five methods for finding conserved sequences in multiple alignments of gene regulatory regions. Nucleic Acids Res.

[Stojanovic2004] Stojanovic N. (2004). Computational methods for the analysis of differential conservation in groups of similar DNA sequences. Genome Inform.

[Stojanovic2009] Stojanovic N. (2009). An improved algorithm for the location of heaviest segments in genomic sequences. Proceedings of the 2009 International Joint Conferences on System Biology, Bioinformatics and Intelligent Computing, IJCBS09..

[TheENCODEProjectConsortium2004] The ENCODE Project Consortium (2004). The ENCODE (ENCyclopedia Of DNA Elements) Project. Science.

[TheENCODEProjectConsortium2007] The ENCODE Project Consortium (2007). The ENCODE pilot project: Identification and analysis of functional elements in 1% of the human genome. Nature.

[Thomasetal2003] Thomas J.W., Touchman J.W., Blakesley R.W., Bouffard G.G., Beckstrom-Sternberg S.M., Margulies E.H., Blanchette M., Siepel A.C., Thomas P.J. (2003). Comparative analyses of multi-species sequences from targeted genomic regions. Nature.

[Wingender2008] Wingender E. (2008). The TRANSFAC project as an example of framework technology that supports the analysis of genomic regulation. Brief Bioinform.

[Yektaetal2004] Yekta S., Shih I., Bartel D.P. (2004). MicroRNA-directed cleavage of HOXB8 mRNA. Science.

[Zody2007] Zody M.C., Stojanovic N. (2007). Comparative genomics: Identifying functional elements through conservation. Computational Genomics: Curr Methods.

